# The infant gut microbiome as a microbial organ influencing host well-being

**DOI:** 10.1186/s13052-020-0781-0

**Published:** 2020-02-05

**Authors:** Francesca Turroni, Christian Milani, Sabrina Duranti, Gabriele Andrea Lugli, Sergio Bernasconi, Abelardo Margolles, Francesco Di Pierro, Douwe van Sinderen, Marco Ventura

**Affiliations:** 1 Laboratory of Probiogenomics, Department of Chemistry, Life Sciences, and Environmental Sustainability, University of Parma, Parco Area delle Scienze 11a, 43124 Parma, Italy; 2 Microbiome Research Hub, University of Parma, Parma, Italy; 3 Departamento de Microbiologia y Bioquimica de Productos Lacteos, IPLA – CSIC, Villaviciosa, Asturias Spain; 4 Instituto de Investigación Sanitaria del Principado de Asturias-ISPA, Oviedo, Spain; 5 Velleja Research, Piacenza, Italy; 6 School of Microbiology & APC Microbiome Institute, University College Cork, Cork, Ireland

**Keywords:** Bifidobacteria, *Bifidobacterium bifidum* PRL2010, Neonates, Microbiome, Probiotics

## Abstract

Initial establishment of the human gut microbiota is generally believed to occur immediately following birth, involving key gut commensals such as bifidobacteria that are acquired from the mother. The subsequent development of this early gut microbiota is driven and modulated by specific dietary compounds present in human milk that support selective colonization. This represents a very intriguing example of host-microbe co-evolution, where both partners are believed to benefit. In recent years, various publications have focused on dissecting microbial infant gut communities and their interaction with their human host, being a determining factor in host physiology and metabolic activities. Such studies have highlighted a reduction of microbial diversity and/or an aberrant microbiota composition, sometimes referred to as dysbiosis, which may manifest itself during the early stage of life, i.e., in infants, or later stages of life. There are growing experimental data that may explain how the early human gut microbiota affects risk factors related to adult health conditions. This concept has fueled the development of various nutritional strategies, many of which are based on probiotics and/or prebiotics, to shape the infant microbiota. In this review, we will present the current state of the art regarding the infant gut microbiota and the role of key commensal microorganisms like bifidobacteria in the establishment of the first microbial communities in the human gut.

## Introduction

The human gastrointestinal tract (GIT) harbors a wide array of microorganisms, which constitute a complex microbial ecosystem, known as gut microbiota, which reaches its highest microbial density in the colon. The gut microbiota is believed to play (a) key role(s) in maintaining and supporting human health, and any disturbance in its composition, sometimes referred to as intestinal dysbiosis, is believed to facilitate the onset of and/or aggravate certain diseases, including autoimmune and allergic diseases, colorectal cancer, metabolic diseases and bacterial infections. Indigestible food components, i.e., prebiotics, as well as the use of health-promoting viable microorganisms, i.e., probiotics, are often used as biotherapeutic agents in order to re-establish/maintain normal homeostasis of the gut microbiota [for a review see [1]].

The extent of microbiota composition and diversity has only recently been made possible through the use of culture-independent based approaches, known as metagenomics, aimed at revealing the presence of microbes based on their DNA sequences. Part of the microorganisms that reside in the intestine can be cultivated [2, 3], though up to 50% of the microbial population is currently considered unculturable [4–6]. The first metagenomics-based survey of the fecal microbiota of healthy humans highlighted the extraordinarily high microbial diversity of this environment with the discovery of a considerable number of phylotypes, many of which did not correspond to cultured strains from bacterial collections [7]. Recently, metagenomic data sets have provided a more precise view of the complexity of the gut microbiota composition [8, 9].

The intestinal microbiota is primarily composed of six microbial phyla including Firmicutes, Bacteroidetes, Actinobacteria, Proteobacteria, Tenericutes and Fusobacteria. Notably, the dominant phyla occurring in the adult microbiota are represented by Firmicutes and Bacteroidetes which together may constitute up to 90% of the total gut microbiota [10]. Nevertheless, these values are different in the infant intestine, where Actinobacteria and specifically the genus *Bifidobacterium* are commonly in the majority [11]. In addition, the adult gut microbiota is more complex in terms of total bacterial numbers as well as encountered diversity of microbial taxa [11, 12]. A similar trend was observed for the gut microbiota of the elderly population [13], where the microbial intestinal communities appear to be less diverse. Collectively, the number of bacterial cells residing in the human intestine was previously thought to be 10 times larger than the total number of human cells [14], yet has recently been re-examined and considerably reduced, revealing a 1:1 ratio [15]. Notably, it has been postulated that the genetic arsenal constituted by the different microbes of the gut microbiota, i.e., the intestinal microbiome, is about 150 fold larger than the human gene complement, with an estimated set of 3.3 million bacterial genes [16]. In addition, bacterial communities display quantitative and qualitative variations due to several other variables such as chemical-physical host factors (e.g., pH, bile acids, transit time and mucus), environmental factors (e.g., nutrients and medication) and microbial factors (e.g., adhesion capability, bacterial enzymes, metabolic strategies) [1]. Another key force shaping the diversity and composition of the gut bacterial community is represented by bacteriophages, i.e. viral predators of bacteria [17, 18].

## Functions driven by the gut microbiota

The gut microbiota exerts a primary role in the breakdown of dietary, complex plant carbohydrates (that are indigestible by host enzymes) and host-produced glycans [e.g., mucin and human milk oligosaccharides (HMOs)], as well as in the protection against pathogenic bacteria. Furthermore, the gut microbiota is supposed to be essential for the correct development of the host immune system [19]. In this context, there are growing indications that the gut microbiota plays an important role in inducing IgA production [20, 21], as well as in preserving the homeostasis of several T cell populations in the gut, comprising regulatory T cells (Treg), T helper 1 (Th1), and 17 (Th17) cells [22], as well as MAIT cells [23].

Further metabolic functions of the gut microbiota encompass bile acid biotransformation, as well as production of vitamins and synthesis of short-chain fatty acids such acetate, propionate and butyrate. Furthermore, other microbial products comprise lactate, ethanol, succinate, valerate, caproate, isobutyrate, 2-methyl-butyrate and isovalerate. The most pronounced effects of these metabolites are their trophic roles toward the intestinal epithelium [24]. In particular, butyrate is the preferred energy source for enterocytes.

Notably, commensal bacteria that normally reside in the gut, i.e. the autochthonous or indigenous gut microbiota [25] are diverse between individuals and specific species are known to possess distinct and in some cases opposing roles. In this context, certain microbiota members have been shown to influence Treg development, whereas others promote Th17 development. Thus, a well-adjusted autochthonous microbiota is essential to drive the normal development of both mucosa-associated lymphoid tissue and mucosal induced tolerance mechanisms based on the generation of Treg cells. Colonization of mice deprived of the gut microbiota, i.e. the germ free mice (GF), with *Bacteroides fragilis* has been demonstrated to enhance the suppressive capability of Tregs to promote anti-inflammatory cytokine production [26]. In a similar fashion, the colonization of GF mice with a mix of *Clostridium* strains increased the production of IL10-producing Tregs in the colonic lamina propria [27]. Besides, many other gut microorganisms have been shown to modulate Treg production, including microbial taxa that are commonly exploited as health-promoting bacteria such as many members of the *Bifidobacterium* genus [28, 29]. Conversely, Th17 cell response in humans was demonstrated to be generated by a particular and recently discovered bacterial group named the segmented filamentous bacteria. Among this latter group of bacteria it is worth mentioning *Candidatus arthromitus*, a microorganism known to colonize the small intestine and capable of modulating IgA synthesis [20, 21]. Recently, *C*. *arthromitus* has been demonstrated to enhance the development of Th17 cells, while it has also been shown to be involved in the etiology of a number of inflammatory and autoimmune diseases. Thus, a microbiota that favors this microorganism has an impact on the immune response and consequently on the development of Th17-mediated inflammatory/autoimmune diseases in the gut and at distal sites in the predisposed individuals. Recently, it has been shown that *Prevotella histicola* modulates the systemic immune responses with the decrease in pro-inflammatory Th1 and Th17 cells and an increase in the frequencies of regulatory T cells, tolerogenic dendritic cells, and suppressive macrophages [30].

## The origin of the gut microbiota

It is generally perceived that the human gut is microbiologically sterile at birth, yet at that point will become subject to extensive bacterial colonization (for review see [18]). However, bacterial colonization is influenced by both the delivery method, i.e. vaginal [31] versus caesarian section [32], and by the feeding system, i.e. breast-milk versus formula-milk [33]. It is believed that the main modifications of the gut microbiota occurring during these first stages of life are attributed to antibiotic treatments, GIT infections, as well as dietary modifications [34], as opposed to being stochastic [35].

Another important variable involved in the establishment/development of the gut microbiota is represented by the host genotype, although current scientific literature on this topic is still very scarce [36–39] making this contribution not yet clearly defined and a subject of debate. The environment is also considered to play a key role in the establishment of the gut microbiota. In this context, the impact of companion animals is worth mentioning as a valuable source of microorganisms that can be transferred to the human host [40].

Dietary effects on the gut microbiota composition and health are associated with variation of host genotypes and environmental exposures. Growing evidences propose that long-term diet is a primary force responsible for shaping the gut microbiota. This hypothesis is, for example, supported by co-evolution studies which have demonstrated that gut microbiota composition and functions are adapted to the diets of their corresponding mammalian host [41, 42].

Other factors that are considered important for the establishment of the infant gut microbiota are those occurring at prenatal stage, such as the mother’s age, length of gestation (i.e. full-term versus pre-term), smoking habits, probiotic use during pregnancy, intrapartum antibiotic therapy, intrauterine environment or body mass index [43, 44].

## Gut microbiota as a possible predictor of the human health

Recently, it has been documented that breast feeding and vaginal delivery exert a positive influence on the health status of the infant that may also impact positively later in life. Breast feeding and vaginal delivery have been associated with lower incidence of immune related disorders. Conversely, early perturbations of the infant gut microbiota, such as those provoked by antibiotic therapies or other microbial stress factors, can lead to chronic metabolic disorders. For instance, the phylogenetic diversity of the newborn gut microbiota increases gradually over time and modifications in community composition correlates with a very regular temporal gradient. In contrast, some taxa show abrupt shifts in abundance corresponding to changes in diet. In fact, although the earliest infant gut microbiota seems to be mainly enriched in genes facilitating lactose and lactate utilization, functional genes involved in adult food processing are already present before the introduction of solid food, priming the infant gut for an adult diet. Therefore, even modest introduction of solid foods, typical during the weaning phase, causes a sustained increase in the abundance of adult phyla (i.e. Bacteroidetes), elevates fecal short chain fatty acid levels and genes associated with carbohydrate utilization, vitamin biosynthesis, and xenobiotic degradation, and initiates the assembly of a more stable community composition. Also health conditions affect diversity, richness and taxa, as observed in case of fever and/or after administering antibiotics for bacterial infection where diversity drops and taxa like *Bifidobacterium* and *Bacteroides* seem to disappear [34, 45]. Apparently, different microbial colonization profiles are responsible for very different physiological consequences on child health [46]. Therefore, knowledge on the factors that shape the developing gut microbiota from birth is critical to develop/establish targeted strategies to minimize the long-lasting effects of early disturbances in the human microbiota [47].

Microbial colonization of the human gut is currently believed to be one of the main determinants for priming and programming of the human immune system, while it is also presumed to drive correct development of the intestinal tract and determine its metabolic activities. These physiological processes are guided by constant communication between members of the gut microbiota and their host. Therefore, intestinal dysbiosis may disrupt microbe-host dialogue, which in turn may trigger long-lasting physiological effects and health disorders [48]. Among these, extensive research has been performed focusing on the potential effects of early life microbiota and immune disorders. In this context, the most convincing data originate from animal (murine) studies, revealing a close relationship between early exposure to microorganisms and development of immune pathologies. Though this association had been recognized for decades, these recent studies have contributed to understand the mechanisms behind the long-term effects of the gut microbiota in influencing the immune response. In this context, it has been shown that microbial factors control the activity of chemokine ligand CXCL16, which modulates the increase of invariant natural killer T cells in the colon and lungs, and that neonatal colonization of GF mice with a conventional microbiota prevents their accumulation [49]. It has been suggested that early-life microbiota imposes long-lasting effects, and that the absence of such a microbial stimulus may induce later-life inflammatory responses reminiscent of those seen in Inflammatory Bowel Disease (IBD) and asthma [49]. The importance of a critical window within which disruption of the intestinal microbiota may have long-lasting consequences in immune pathologies has been suggested, and the ordered establishment of appropriate cross-talk between gut commensals and the intestinal mucosal surfaces has been highlighted. This cross-talk is facilitated by host-microbial interactions that are already believed to occur immediately following birth, suggesting that disease risk may be affected during early life [50].

### Atopic diseases (asthma and atopic eczema)

Among the Th2-mediated immune pathologies that are linked to a particular microbiota establishment, atopy, mainly in the form of asthma and atopic eczema, has been associated with specific microbial features occurring during the first days following birth.

There are several epidemiological studies suggesting that early development of the infant gut microbiota affects the risk of asthma at later stages in life [51]. This has been linked to an inappropriate development of the gut microbiota and the (associated) disturbance of immune homeostasis during the first year of life [52]. Nevertheless, there is no scientific data showing a direct association of specific microbial patterns in the early stages of life with the development of asthma in later life, since genetic, epigenetic and other environmental factors also affect the development of this disease. However, it is becoming increasingly clear that the gut microbiota exploits an important role in the perinatal programming of asthma [53]. In this context, recent evidence indicates that the risk of suffering from asthma is higher in infants exhibiting a gut microbiota dysbiosis during the first 100 days of life, and that such risk has been associated with the presence of particular microbial groups [18]. In this regard, a recent study assessing the gut microbiota composition of 319 subjects of a Canadian cohort, showed that infants at high risk of developing asthma possess a significantly decreased relative abundance of certain microbial genera such as *Lachnospira*, *Veillonella*, *Faecalibacterium* and *Rothia*. In addition, such differences in the abundance of bacteria taxa were linked to levels of fecal bacterial metabolites. Inoculation of these bacteria in GF mice reduced the airway inflammation in their progeny, suggesting that these bacteria possess a pivotal role in the etiology of asthma [54]. Moreover, low gut microbiota diversity during the first month of life has been linked with a higher prevalence of asthma in 7 years old children [55]. Similarly, lower levels of *Lachnospira* and higher loads of *Clostridium* spp. at 3 months have been positively linked with asthma risk by 4 years of age, suggesting that the ratio between these two bacteria could be used as a biomarker for asthma development [56].

Chronic recurring atopic eczema is the principal clinical sign for atopic disease during the first years of life [57]. The association between the gut microbiota and atopic eczema has been established through the use of a culture-independent PCR-based approach, which revealed differences in the gut microbiota composition at the age of 1 month, a period that precedes the manifestation of atopic symptoms within the first 2 years of life. Specifically, the occurrence of *Escherichia coli* has been linked with a higher risk of developing eczema, and the presence of *Clostridium difficile* was associated with several atopic outcomes, including eczema, wheeze and allergic sensitization [58]. Such findings were confirmed in another large study involving 646 infants, in which an association was detected between colonization by *E. coli* and development of IgE-associated eczema within the first year of life [59]. Subsequent studies indicate that a reduced microbial diversity of early life microbiota directly correlates with later development of atopic eczema [60–62]. Interestingly, it has recently been highlighted that the butyrate-producing bacteria *Coprococcus eutactus* is implicated in the reduction of the severity of eczema [63]. Although these data do not associate a particular early microbiota profile with a decrease of atopic eczema ailment, it nonetheless indirectly points to the potential protective role of butyrate-producer bacteria against the development of atopic eczema. Notably, another study pointed out that a low load of butyrate-producers appears to commonly precede the development of atopic eczema [64].

As previously underlined, low microbial diversity during the early stages of life and/or the lack of particular microorganisms may play a critical role in the subsequent development of allergic diseases. This has been highlighted by a recent study where a correlation between the absence or the reduction of taxa like *Bifidobacterium*, *Akkermansia* or *Faecalibacterium* and childhood atopy, and asthma, was observed [65]. Thus, there is a “window of opportunity,” aligning with the establishment of an early microbial colonization, where microbiota modulation may prevent disease, for example through the use of probiotics. In this context, it has been shown that babies that were colonized with *Bifidobacterium breve* during the early post-natal period (1 week and 3 months) displayed a reduced risk of developing eczema in the first year of life [66]. A small number of innovative studies reported a long-term prevention of atopic eczema by probiotics provided to mothers during pregnancy or postnatally to infants [57, 67, 68]. However, caution should be taken in the biological significance of these results since some of these findings could not be reproduced with different cohorts though employing the same probiotic strains [69]. In relation to this, considering the scientific evidence generated in various human clinical trials using probiotics, the World Allergy Organization has recently published recommendations about the use of probiotics in the prevention of allergy. These guidelines concluded that, although current evidence likely supports a net benefit related to the use of probiotics in eczema prevention, there is not enough evidence supporting the recommendation of probiotics for the reduction of allergy risk in children [70].

### Food allergy

Intestinal bacteria are critical for regulating allergic responses to dietary antigens and this suggests that interventions modulating bacterial communities are therapeutically relevant for food allergy. According to a very recent study, the presence of a Clostridiales taxa *Anaerostipes* (i.e., in particular the species *Anaerostipes caccae*) has been associated with a reduction of the risk of food allergy in infant [71]. Furthermore, gut microbiota composition seems to play a role in food allergy resolution in infants suffering from cow’s milk protein allergy [72].

### Metabolic disorders

Gut microbiota composition and function has been linked with obesity and obesity-related disorders. By enhancing energy harvest, the so-called obesogenic microbiota modulates obesity behavior and peripheral metabolism. It has been indicated that different factors influencing the establishment of the gut microbiota during infancy are responsible for obesity during subsequent stages of life [73]. Microbiota-related obesity studies in animal models, particularly in rodents, have increased our understanding of the role of gut microbiota in driving metabolic disorders. Nevertheless, in recent years it has become clear that extrapolation of results of animal studies to humans comes with a lot of caveats, highlighting the need to confirm such results by human clinical trials [74]. It has been suggested that early microbial patterns may predict overweight in children [75]. In this regard, a large cohort study, involving 909 one-month-old infants, revealed that levels of *Bacteroides fragilis* at 1 month of age are significantly associated with a higher Body Mass Index (BMI) in children [76].

Appropriate conservation of the intestinal barrier function seems to be pivotal for an adequate metabolic balance. Thus, different factors disturbing the microbial climax during early life play a key role in overweight, obesity development and child adiposity in later life. Among these factors, nutrition, maternal obesity, delivery mode, intestinal permeability, pathogenic infections or antibiotic use, have been highlighted [77–80]. Furthermore, the impact of the gut microbiota on brain developmental programming of obesity has also been suggested [81].

Dietary manipulation with probiotic formulations holds promise to prevent obesity [82]. However, in general, clinical studies on a direct impact of probiotic interventions on weight development are very scarce, and further epidemiological studies and clinical trials are needed in order to obtain solid scientific evidence supporting the use of probiotics in obesity prevention [83].

There is growing interest concerning the role of early-life antibiotic therapy in the development of metabolic diseases. In this regard, many murine studies have indicated that gut microbiota perturbations caused by antibiotics during the establishment of the early infant gut microbiota has long-lasting metabolic consequences, including adiposity, insulin resistance, type 2 diabetes and liver disease [84–86]. Epidemiological studies in humans also demonstrated that antibiotic exposure is associated with long-term metabolic effects including obesity in children and adults. Studies with large infant cohorts highlighted a link between early live antibiotic exposure and childhood obesity [87]. Furthermore, it has been suggested that disturbances of the intestinal microbiota caused by antibiotics, either during prenatal and postnatal periods, may increase the risk of becoming obese [88, 89]. However, further evidences from epidemiological studies are necessary to establish a definitive link between early life antibiotic-induced dysbiosis and the subsequently development of metabolic disease.

### Other diseases

Dysbiosis occurring during the early stages of life has also been related with other long-lasting effects, such as Inflammatory Bowel Disease (IBD), Irritable Bowel Syndrome (IBS) and type 1 diabetes (T1D).

T1D is an autoimmune disease caused by the destruction of insulin-producing beta cells. T1D may influenced by several environmental factors, and the gut microbiota has been put forward as a possible instigator in the development of this disease. Longitudinal studies in patients with T1D have revealed a lower diversity and significant differences in the ratio of Firmicutes and Bacteroidetes, as well as a significant reduction in the load of the butyrate-producer *Faecalibacterium prausnitzii* in diabetic children [90]. Indeed, butyrate-producing bacterial species are more abundant in non-diabetic children, and it has been suggested that these bacteria play a key role in reducing the risk of developing T1D [91]. Furthermore, how early intestinal colonization can impact on the subsequent progression of T1D has also been studied. Notably, it has been shown that microbiota perturbations during early infancy may establish a pro-inflammatory environment promoting the development of autoimmune disease [92].

Diseases involving intestinal inflammatory processes have also been related to early microbiota colonization in infants. The fact that the human intestinal immune system is educated during the first stages of life by the microbial colonization has clearly defined a link between the pioneering gut microbial inhabitants, such as bifidobacteria, and subsequent intestinal inflammatory disease [93–96]. However, data about the long-term effects of early dysbiosis in the development of IBD and IBS in childhood and adults are currently lacking and do not allow the establishment of causality. Thus, future work involving animal models and human epidemiological studies should be aimed at revealing microbe-mediated immune and physiological responses that prevent or promote these diseases.

## The gut microbiota of infants and the threat of neonatal pathologies

The neonatal stage can be characterized by a high risk of nosocomial infections, which are commonly explained by an immune system that it is still immature in early life. Such disease issues are highly exacerbated in preterm infants, where the frequent use of catheters commonly trigger infections by *Staphylococcus* spp., as well as *E. coli* and *Klebsiella* spp. [97–99]. In pre-term infants this sepsis risk is aggravated, as well as the risk of developing Necrotizing Enterocolitis (NEC), representing a very serious and frequently fatal disease [100]. In this context, neonates developing NEC will also suffer from sepsis, generally generated by a dominant member of the gut microbiota, such as enterobacteria, of such pre-term infants [98, 99].

So far, the etiology of NEC is still unknown, although particular microorganisms have been proposed as causative agents [101]. Notably, it has been shown that the gut microbiota of infants who develop NEC possesses a reduced bacterial diversity while at the same time containing enhanced levels of potentially pathogenic microorganisms [102, 103]. Nevertheless, the clinical trials that have been performed have not yet identified a clear microbial profile of dysbiosis, though such studies have frequently detected a high load of Proteobacteria preceding NEC establishment [104–107]. In addition, a markedly reduced level of bifidobacteria has been observed and consequently the lack of their potential protective role [108]. Specific metabolic pathways associated with NEC have been described [100] and metabolome deviations have been observed in the serum of these infants [108].

As a general interpretation of the possible link between NEC and gut microbiota, one may argue that an increased immune response triggered by a high load of intestinal Proteobacteria enhances the risk of bacterial translocation and sepsis [109]. Many metagenomic-based studies have highlighted a lower microbial diversity and low levels of *Bifidobacterium* and *Bacteroides,* with a predominance of enterobacteria, in infants who develop late onset sepsis as compared with healthy counterparts [110–112]. Nevertheless, available scientific evidence is still limited and further investigations are needed before drawing conclusions on the role that early microbiota alterations play in determining the risk of infection and sepsis.

## The core infant gut microbiota

The infant gut microbiota displays low diversity and its structure is generally unstable and highly dynamic [113]. Conversely, the intestinal microbiota of adults is specific to an individual and relatively stable [13]. However, bifidobacteria are frequently detected in high amounts in infants and consequently are considered a corner stone member of the infant gut microbiota [11, 113–115].

The infant gut microbiota has by some authors been classified into six microbiota types or groups, according to the composition of the gut microbiota within which certain bacterial taxa are abundantly present [18, 116]. Such infant gut microbiota groups encompass: Group 1 consisting of Enterobacteriales; Group 2 involving Bacteroidales and Verrucomicrobiales; Group 3 encompassing all Selenomonadales as well as the Clostridiales genera *Pseudoflavonifractor* and *Subdoligranum* and the inter-individual level and delta-Proteobacteria *Desulfovibrio*; Group 4 including all Pasteurellales; Group 5 comprising most of the Clostridiales and Group 6 involving the Clostridiales genera *Anaerostipes* and *Faecalibacterium*, the Lactobacillales and Bifidobacteriales [116]. Even though bifidobacteria are part of just one of the above mentioned six groups, at the abundance level they represent, together with *Veillonella, Streptococcus*, *Citrobacter, Escherichia,* as well as *Bacteroides* and *Clostridium*, the microbial genera that dominate the infant core gut microbiota [116].

## Bifidobacteria as the key microbial group of the infant gut microbiota

Bifidobacteria belong to the Actinobacteria phylum, which were first isolated from feces of a breast-fed infant by Tissier in 1899, and then named *Bacillus bifidus* [117]. Bifidobacteria are widely distributed among animals whose offspring enjoy parental care, such as mammals, birds, social insects [118]. Consequently, the cause for this intriguing ecological distribution may be explained to direct transmission of bifidobacteria from mother/carer to offspring [119, 120]. Bifidobacteria have also been retrieved in three other ecological niches, including human blood (*Bifidobacterium scardovii*), as well as sewage (e.g. *Bifidobacterium minimum* and *Bifidobacterium thermacidophilum)* and food products (e.g. *Bifidobacterium animalis* subsp*. lactis*). Nevertheless, some caution should be observed in the biological significance of these “atypical” ecological origins, since it is plausible that the identification of bifidobacteria in these environments is due to fecal contaminations [121].

Amongst the 80 (sub) species bifidobacteria so far recognized by the International Commission of Bacterial Taxonomy [122], it is possible to identify bifidobacterial species which elicit a rather broad host species preference, i.e., engaging in a cosmopolitan lifestyle [122]. Intriguingly, *Bifidobacterium bifidum, Bifidobacterium breve* and *Bifidobacterium longum* are specifically identified in the human gut and have been shown to represent part of the dominant bacterial members of the gut microbiota of breast-fed infants [120, 123].

## Bifidobacterial population in the human gut

Bifidobacteria rapidly colonize the large intestine of infants during the first weeks of life, believed to be at least partly due to selection by breast or formula milk, as confirmed by metagenomic analyses [34, 114]. In breast-fed infants, *B. breve* is the dominant species, followed by *B. bifidum*, *B. longum* and *B. adolescentis* [11, 124].

The fecal microbiota of infants is generally characterized by high levels of bifidobacteria [11, 125], whose load decreases after weaning and aging, though ecological analyses based on Fluorescence in situ hybridization (FISH) and metagenomic studies have estimated that their presence in the adult large intestine could reach 4.3 ± 4.4% of fecal microorganisms [12, 126]. It has been estimated that the bifidobacterial communities of the adult colon are dominated by *B. adolescentis* as well as *B. catenulatum* and *B. longum* subsp. *longum* species [127, 128]. Notably, metagenomics as well as culturomic approaches showed that each subject possesses a specific population of colonic bifidobacteria that is in agreement with the large inter-variability of the whole intestinal microbiota previously described [12, 35]. In addition, cataloguing of bifidobacterial communities of both the infant and the adult human led to the identification of many novel bifidobacterial 16S rRNA gene sequences, which are presumed to represent as yet undefined novel bifidobacterial species [120, 128].

As mentioned above, *B. bifidum* is among the first colonizers of the human gut, reaching high numbers in the infant gut, but also detected at very low levels in adults [11]. Thus, *B. bifidum* species is considered a key member of the human intestinal bifidobacterial population and of the infant core microbiota of infants.

## The case of *Bifidobacterium bifidum* PRL2010 as an intriguing example of a microbial inhabitant of the infant gut microbiota

As described above *B. bifidum* was the first bifidobacterial species to be characterized at the beginning of the last century by Tissier [129], having been isolated from stool samples of a breast-fed infant. Since then a growing number of bifidobacterial species have been identified and characterized, with some of these, in particular *B. bifidum* strains have been shown to be dominant in the infant gut [120] and predicted to be vertically transmitted from mothers to corresponding children [120, 130]. In this context, in 2010 a *B. bifidum* strain, named PRL2010, was isolated from the fecal sample of a 3 month-old, Italian, healthy, breast-fed infant. Since then, many scientific publications related to this strain have been generated, providing a very detailed characterization of its ecology, physiology and genetic features, in addition to its cross-talk activities with its host and other members of the infant gut microbiota [118, 131–148].

### The ecological role of PRL2010 within the infant gut microbiota

As described above bifidobacteria constitute one of the first microbial colonizers of the infant gut [18]. Remarkably, the human gut at birth is assumed to be a microbiologically sterile environment, which is exposed to bacterial colonization of microorganisms, such as bifidobacteria, that are maternally acquired by vertical transmission including direct mother-baby contact at birth yet also through breastfeeding [18]. Nevertheless, in case of “non-natural” settings like a Caesarian delivery or bottle feeding, the large intestine of a baby may be prone to aberrant bacterial colonization by environmental microorganisms including pathogens that may provoke long lasting health effects on the host. Interestingly, a probiotic intervention at this stage of life may be crucial in terms of preventing the establishment of a dysbiotic microbiota with its associated negative health implications.

In this context, it has been shown that *B. bifidum* PRL2010 exert an altruistic syntrophic effect to benefit nutrients to the gut microbiota, particularly with respect to other members of (healthy) infant-associated bifidobacterial communities [144]. In vivo murine trials involving *B. bifidum* PRL2010 revealed that, in contrast to other bifidobacterial strains like *B. longum* subsp. *intantis* ATCC15697, PRL2010 possesses cross-feeding features that support growth of other bifidobacteria [144]. The cross-feeding characteristics of *B. bifidum* PRL2010 were further evaluated through in vitro assays aimed at exploring how co-cultivation of PRL2010 cells with other bifidobacterial strains belonging to various species enhanced growth abilities of these latter as compared to growth yields achieved when these bacterial strains were cultivated on their own. This interesting biological feature of *B. bifidum* PRL2010 was observed when this strain was cultivated on host-glycans like mucin and HMOs [132, 133] and on plant-derived carbohydrates like starch and xylan [143]. It has been shown that *B. bifidum* PRL2010 releases rather simple glycans from these complex carbohydrates so that these become accessible to other members of the (bifido) bacterial community. Conversely, the latter bacteria do not possess the necessary enzymatic arsenal to directly metabolize these complex carbohydrates by themselves.

The occurrence of *B. bifidum* PRL2010 cells in the cecum of mice has been shown to promote an expansion of the murine intestinal glycobiome, representing the complete genetic arsenal requested for the metabolism of carbohydrates, toward enzymatic breakdown of both plant- and host- derived carbohydrates [144]. Remarkably, *B. bifidum* PRL2010 modulates the murine gut microbiota by an increase of those microbial groups responsible of the production of Short Chain Fatty Acids (SCFA) such as butyrate and propionate [144].

Another interesting cross-talk activity between *B. bifidum* PRL2010 and other microbial members of the human gut microbiota is represented by the ability of this strain to synthetize extracellular proteins, known as sortase-dependent pili, particularly when it is residing in the mammalian gut [118, 135]. Interestingly, these proteinaceous appendages, apart from playing an important role in driving the interaction of bacteria with the host (see below), also appear to be crucial in modulating physical contacts with other bifidobacterial cells and other human gut commensals through the establishment of cell aggregation [141].

### Genetic adaptation of *B. bifidum* PRL2010 to the human gut

The human gut mucosa is covered by a thick mucin layer, which provides protection against injury and prevents penetration by intracellular pathogens. Furthermore, mucin may exploit a prebiotic role by stimulating the growth of particular members of the autochthonous gut microbiota [145]. The breakdown of mucin, which is expected to diminish the thickness of the mucin layer and consequently decrease the protective barrier covering the intestinal mucosa, may be considered as an undesired event. However, one may also consider degradation as an evolved “host-settler mechanism”. In this context, the production of mucin in the human colon generally only begins several months following birth, reaching its full synthesis level at about 1 year of age [149]. Notably, mucin breakdown as driven by certain human gut commensals, including *B. bifidum* may consequently trigger the secretion of additional colonic mucin, thus recovering or even increasing the thickness of the total load of mucus layer present on the mucosa, thereby reinforcing the epithelial barrier function, which represents an important feature especially in those subjects affected by irritable bowel syndrome [150].

Amongst the bifidobacterial communities, strains belonging to the *B. bifidum* species are currently the only bifidobacteria that are known to actively metabolize and actually grow on mucins [146, 151]. Genome sequencing of *B. bifidum* PRL2010 combined with functional analyses such as proteome and transcriptome analyses, allowed to precisely define the genetic determinants responsible for the metabolism of these host-glycans [146]. According to the Carbohydrate Active Enzymes (CAZy) system, the genome of *B. bifidum* PRL2010 encodes members of two carbohydrate-binding module (CBM) families, CBM32 and CBM51, which were predicted to link to carbohydrate residues encountered in the mucin core structure [152]. Notably, among all assessed bifidobacteria such CBM32 and CBM51 encoding modules were identified only in the chromosomes of *B. bifidum* [145].

Chemically similar host-glycans are represented by HMOs, which are consequently efficiently degraded by many members of the *B. bifidum* species, including PRL2010 [123, 146].

In addition, in depth investigation of the genetic arsenal needed for the uptake of carbohydrates of *B. bifidum* PRL2010 highlighted rather modest carbohydrate transport abilities, which may suggest an interesting genetic strategy for efficient colonization and survival of this strain in the infant gut [140].

Another key example describing the genetic adaptation of *B. bifidum* PRL2010 to the human intestine is constituted by the ability of *B. bifidum* PRL2010 cells to adhere to human cell lines like Caco2 and HT29 monolayers [136]. Notably, the adhesion index of *B. bifidum* PRL2010 cells to these human monolayers is considerably higher as compared to other extensively exploited bifidobacterial probiotic strains like *Bifidobacterium animalis* subsp. *lactis* BB12 [136]. The molecular mechanism driving this intriguing behavior of *B. bifidum* PRL2010 has been discovered [135], being represented by the production of sortase-dependent pili modulating both adhesion to human enterocytes through extracellular matrix (ECM), and being considered crucial for microbe-host cross-talk [135, 153]. Remarkably, pilus production appear to be enhanced when *B. bifidum* PRL2010 cells are located in their natural ecological niche, i.e., the human gut, or when this habitat is simulated under in vitro conditions, e.g., by cultivating PRL2010 in the presence of complex carbohydrates commonly found in the human large intestine [135, 141].

The pili of *B. bifidum* PRL2010 cells have also been reported to modulate innate immunity of the host [135, 138]. According to the latter work, pili of *B. bifidum* PRL2010 are implicated in the developmental programming of the host immune system during infancy, being involved in the imposition of barriers that specifically act against possible bacterial infections.

Other extracellular structures of *B. bifidum* PRL2010 cells such as teichoic acids, represent possible candidates for promoting the interaction of this strain with the host [147]. However, these data are merely supported by comparative genome analyses and further in vivo validations of their possible host interaction activities are therefore needed.

### The interaction of *B. bifidum* PRL2010 with the host immune system

It has already been established that members of the *B. bifidum* species have been playing essential roles in the evolution and maturation of the immune system of the host, which is immature in neonates [154]. It has been shown that *B. bifidum* strains, including PRL2010, in contrast to other bifidobacterial taxa, may cause significantly enhanced production of cytokine IL-17 [146, 154]. The interaction of *B. bifidum* PRL2010 cells with the host immune system has been investigated in considerable detail through the use of both an in vitro cell line- and a murine- model [138]. Notably, the overall host-response scenario driven by *B. bifidum* PRL2010 cells can be explained as a local pro-inflammatory response, modulating the immune system. Simultaneously, *B. bifidum* PRL2010 cells trigger an attenuation of the pro-inflammatory response by down-regulating particular chemokines like the heat shock proteins, while up-regulating defensin and tight junction genes [138]. In addition, ELISA experiments demonstrated that exposure to *B. bifidum* PRL2010 cells activates synthesis of IL-6 and IL-8 cytokines, presumably through NF-kb activation [138].

The roles of PRL2010 on the mucosal integrity have been evaluated through an in vivo murine model simulating an ulcerative colitis status [138]. In the latter study, the pretreatment with *B. bifidum* PRL2010 cells was shown to elicit increased expression of tight junction-encoding genes, being associated with a marked reduction of all colitis-associated histological parameters [138].

## Conclusions

Genetic and environmental factors are known to modulate the composition of the gut microbiota, which is believed to exert key roles in modulating the immune response at both intestinal and extra-intestinal sites, as well as in controlling the development of certain types of autoimmune and allergic diseases and particular types of cancers. The relationship between gut microbiota, immunity and disease is very intricate, since the same commensal bacteria can induce either a protective response or a pathogenic/inflammatory response, depending on the susceptibility of the individual. Notably, despite numerous efforts the specific microorganisms contributing to either the aetiology and/or protection from different types of diseases, i.e. those microbes that could therefore be employed as microbial biomarkers, have not yet been fully characterized. However, in the case of the infant gut microbiota, it has been demonstrated that a drastic reduction of its complexity and a reduction of the bifidobacterial load is commonly associated with diseases and or disorders that are established later in life [18]. Thus, precise evaluation of the intestinal microbiota composition of the newborn will in the not too distant future become a valuable clinical procedure in order to predict and possible prevent the development of diseases/disorders.

The discovery of microbial biomarkers and effective prebiotic/probiotic compounds will be crucial in order to establish biotherapeutical protocols for the prevention and treatment of gut microbiota-mediated diseases. There is a growing interest by the scientific community in the exploitation of a next generation of probiotic bacteria, i.e., those whose health-promoting activities are supported by rigorous scientific/genetic proof and possessing the ability to modulate the gut microbiota. Such probiotic applications may be highly effective in environments where the natural microbial complexity is rather simple, such as the infant gut microbiota. There is a growing scientific literature describing the use of probiotics for the treatment of acute gut infectious diseases, in necrotizing enterocolitis, acute respiratory infections and recurrent urinary tract infections. However, there is still a large gap of knowledge about the molecular basis supporting these potential beneficial effects of probiotic bacteria as well as about the adequate dosages, duration of treatment and safety of such probiotic formulations [155].

Apart from the use of probiotic strains, such as *B. bifidum* PRL2010, for infants, another potential application of these probiotic bacteria includes the treatment of women during pregnancy. Such probiotic therapy may be crucial in order to colonize the mother’s gut by prior the delivery and thus to guarantee the settling of an appropriate bifidobacterial community that will be vertically transmitted to the newborn at birth. Nevertheless, rigorous clinical testing involving these bacteria need to be performed before meaningful probiotic claims (for humans) can be made.

## Data Availability

Data sharing not applicable to this article as no datasets were generated or analyzed during the current study.

## Abbreviations

BMI: Body Mass Index
CAZy: Carbohydrate Active Enzymes
CBM: carbohydrate-binding module
ECM: extracellular matrix
FISH: Fluorescence in situ hybridization
GF: germ free
GIT: gastrointestinal tract
HMOs: human milk oligosaccharides
IBD: Inflammatory Bowel Disease
IBS: Irritable Bowel Syndrome
NEC: Necrotizing Enterocolitis
SCFA: Short Chain Fatty Acids
T1D: type 1 diabetes
Treg: regulatory T cells
